# Supplementary material for: Inner nuclear protein Matrin-3 coordinates cell differentiation by stabilizing chromatin architecture
Source: Nat Commun. 2021 Oct 29;12:6241. doi: 10.1038/s41467-021-26574-4 (PMC8556400; doi:10.1038/s41467-021-26574-4)
Supplement: Supplementary file 1 — Supplementary Information [file 41467_2021_26574_MOESM1_ESM.pdf]

Supplementary Information

Inner nuclear protein Matrin-3 coordinates cell differentiation by stabilizing chromatin architecture

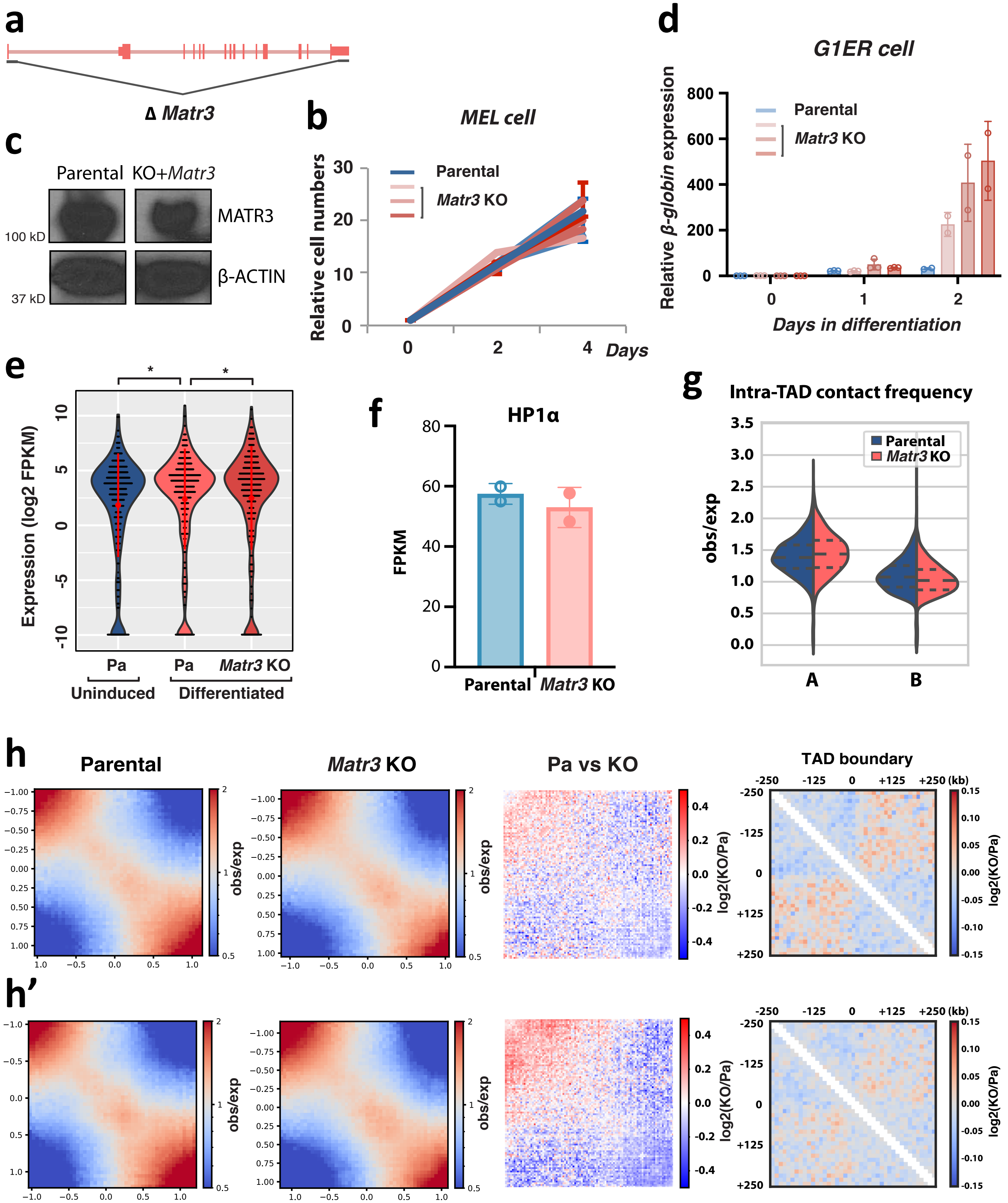

a
Δ Matr3
b
MEL cell
Parental
Matr3 KO
Relative cell numbers
Days
c
Parental KO+Matr3
MATR3
β-ACTIN
100 kD
37 kD
d
G1ER cell
Parental
Matr3 KO
Relative β-globin expression
Days in differentiation
e
Expression (log2 FPKM)
Pa
Pa
Matr3 KO
Uninduced
Differentiated
f
HP1α
FPKM
Parental
Matr3 KO
g
Intra-TAD contact frequency
obs/exp
Parental
Matr3 KO
A
B
h
Parental
Matr3 KO
Pa vs KO
TAD boundary
obs/exp
log2(KO/Pa)
(kb)
h'

Figure S1

Figure S1. ***Matr3*** **knockout cells proliferate normally but display accelerated cell maturation.** (a) *Matr3* gene structure. The entire gene body was deleted by CRISPR/Cas9-mediated gene editing using [1]. (b) Cell proliferation was examined in parental and three independent *Matr3* KO MEL clones. Error bars represent mean ± 1 s.d. across 3 (parental) or 5 (*Matr3* KO) independent experiments. (c) Matr3 expression in parental cells and *Matr3* KO cells rescued with full-length Matr3 cDNA is shown by Western blot. β-actin was used as a control. Data were the result of 2 independent experiments. (d) The expression level of *β-globin* mRNA in G1ER cells was quantified during differentiation. Three independent *Matr3* KO G1ER clones by CRISPR/Cas9 were selected for the experiment. Error bars represent mean ± 1 s.d. across three independent experiments on days 0 and 1, and two independent experiments on day 2. (e) Fragments per kilobase per million reads mapped (FPKM) from RNA-seq of parental and *Matr3* KO cells were compared with mean FPKM values of an erythroid gene list[2]. The expression level was increased during normal erythropoiesis, and notably higher in *Matr3* KO cells (mean values are 32.18112, 42.51672 and 49.45524, respectively; p = 1.73e-02, p = 2.82e-02, respectively, by two-sided t test). (f) HP1α expression level was compared in parental and *Matr3* KO cells using RNA-seq data obtained from two independent experiments. Error bars represent mean ± 1 s.d. (g) The mean contact frequency between the bins within each TAD was calculated to determine the compartment changes within TADs. In *Matr3* KO, intra-TAD contact frequency was increased in compartment A while decreased in B (median values are 1.38, 1.44, 1.07, and 1.02, respectively; p = 2.7e-6, p = 4.6e-10, by

two-sided Mann Whiteney U test, respectively). (h-h') Results of replicate experiments for Figure 2f-h are shown. Source data are provided as a Source Data file.

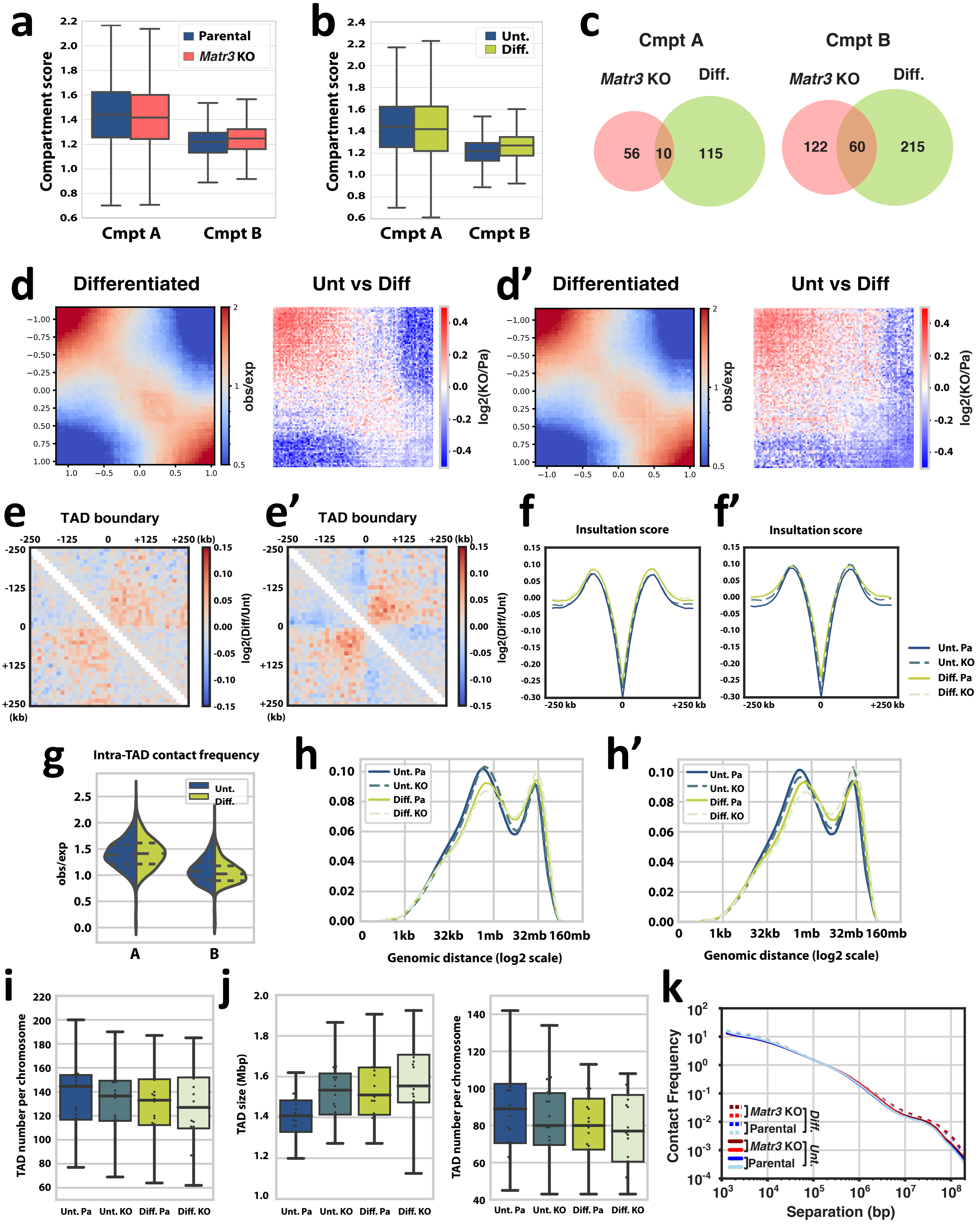

a
Compartment score
Parental
Matr3 KO
Cmpt A
Cmpt B
b
Unt.
Diff.
c
Cmpt A
Cmpt B
Matr3 KO
Diff.
56
10
115
122
60
215
d
Differentiated
Unt vs Diff
obs/exp
log2(KO/Pa)
d'
e
TAD boundary
log2(Diff/Unt)
e'
f
Insultation score
f'
Unt. Pa
Unt. KO
Diff. Pa
Diff. KO
g
Intra-TAD contact frequency
obs/exp
A
B
h
Genomic distance (log2 scale)
h'
i
TAD number per chromosome
j
TAD size (Mbp)
k
Contact Frequency
Parental
Separation (bp)

Figure S2

Figure S2. Changes in chromatin structure during differentiation are similar to those in cells lacking Matr3. (a-b) To further confirm changes in compartment strengths, we used a metric named compartment score[3]. Changes in compartments A (n = 1675) and B (n = 1654) upon *Matr3* loss (a) and during differentiation (b) were assessed by two-sided Mann-Whitney U test (p = 7.7e-11, p = 1.8e-156, p = 5.2e-8, p < 1e-300, respectively). (c) TADs with significantly altered interactions in *Matr3* KO (Figure 2i) and during differentiation (Figure 3c) were compared. TADs with increased interaction in compartment A significantly overlapped (2.03 fold over enriched compared to expected, p < 1e-100, by one-sided hyper-geometric test), and TADs with decreased interaction in compartment B also overlapped significantly (1.98 fold over enriched compared to expectations, p < 1e-100, by one-sided hyper-geometric test). (d-f') Results of replicate experiments for Figures 3a-b and 3d-e are shown. (g) The mean contact frequency between the bins within each TAD was calculated (median values are 1.38, 1.41, 1.07, and 1.02, respectively; p = 0.01, p = 1.2e-7, by two-sided Mann Whiteney U test, respectively; n = 1675, n = 1654, respectively). (h-h') Results of replicate experiments for Figure 3f are shown. (i) The number of TADs for each chromosome is depicted using [4] (Unt.Pa vs. Unt.KO: p = 0.003, Unt.Pa vs. Diff.Pa: p = 0.0003, Diff.Pa vs. Diff.KO: p = 0.31, by two-sided Wilcoxon signed-rank test, respectively; Cohen's d = 0.15, Cohen's d = 0.26, Cohen's d = 0.03, respectively). (j) Average TAD size and the number of TADs for each chromosome is depicted using [5] across two independent experiments (Left, Unt.Pa vs. Unt.KO: p = 1.9e-5, Unt.Pa vs. Diff.Pa: p = 4.5e-7, Diff.Pa vs. Diff.KO: p = 0.88; Right, Unt.Pa vs. Unt.KO: p = 0.0002, Unt.Pa vs. Diff.Pa: p = 1.5e-6, Diff.Pa vs. Diff.KO: p = 0.85, by two-sided Wilcoxon

signed-rank test, respectively). (k) Genome-wide contact probability calculated from Hi-C data. In box plots, center lines, boxes, and whiskers represent the median value, first and third quartiles, and 1.5 interquartile range of the samples, respectively.

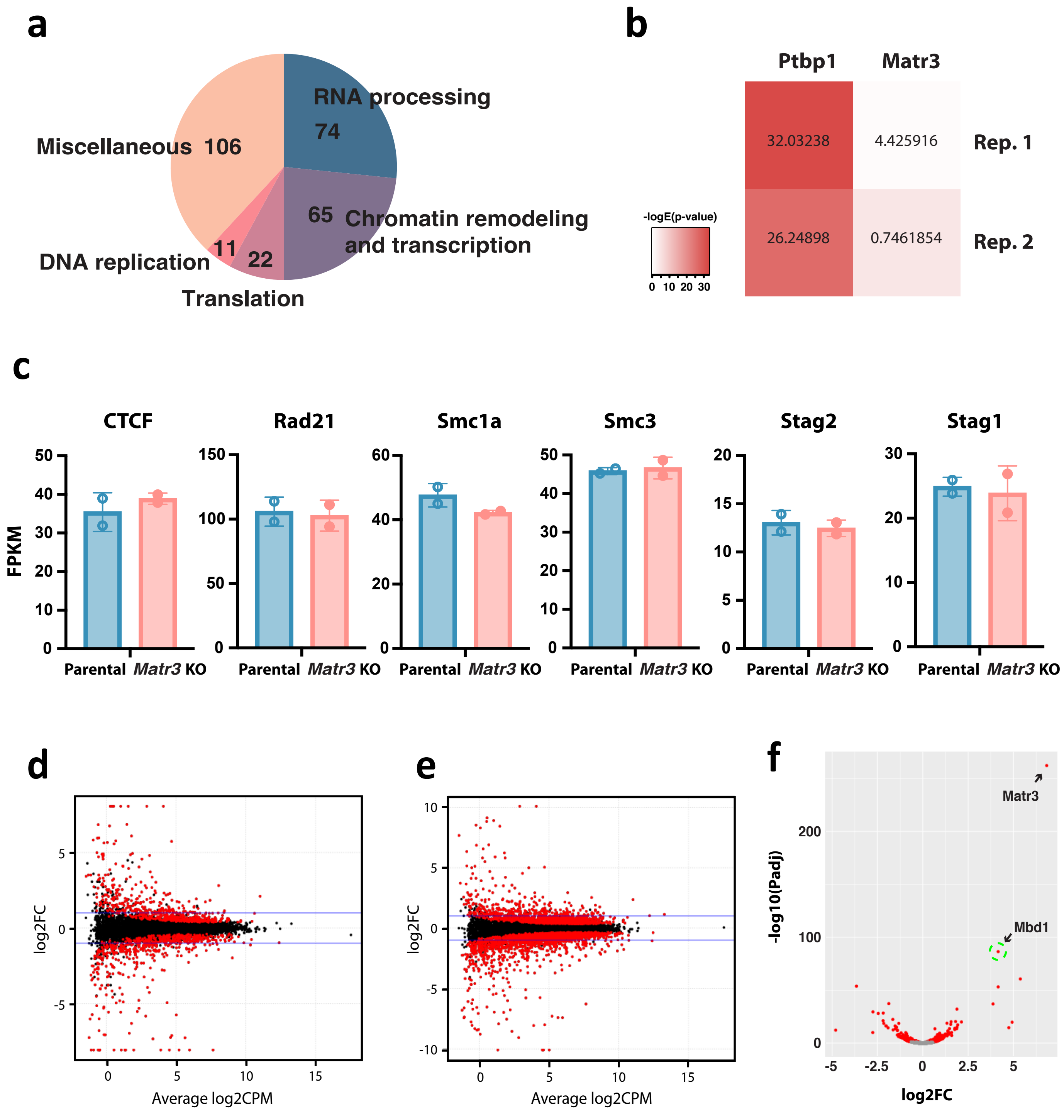
a
RNA processing
74
Miscellaneous 106
65
Chromatin remodeling
and transcription
DNA replication
11
22
Translation
b
Ptbp1
Matr3
32.03238
4.425916
Rep. 1
26.24898
0.7461854
Rep. 2
-logE(p-value)
0 10 20 30
c
CTCF
Rad21
Smc1a
Smc3
Stag2
Stag1
FPKM
Parental
Matr3 KO
d
e
log2FC
Average log2CPM
f
Matr3
Mbd1
-log10(Padj)
log2FC

Figure S3

Figure S3. Matr3 interacts with proteins involved in RNA processing and chromatin remodeling, and changes in gene expression in *Matr3* KO were greater as cell differentiation proceeded. (a) Among the proteins in which at least two total peptides were identified from the IP-MS analysis (Figure 4a), 281 proteins with known functions were classified into the indicated categories. (b) Differential alternative splicing isoforms in *Matr3* KO compared to parental cells were identified using [6], and the association to differentially expressed genes was calculated using the one-sided Fisher's exact test. RNA-seq data obtained from knockdown experiments of a splicing regulator Ptbp1[7] were used for comparison. The results of two independent experiments are shown. (c) Gene expression was compared in parental and *Matr3* KO cells using RNA-seq data obtained from two independent experiments. Error bars represent mean ± 1 s.d. Differentially expressed genes in *Matr3* KO compared to parental cells under uninduced (d) and differentiated (e) conditions. Significantly different genes identified using [8] are shown in red. (F) Differentially expressed genes in *Matr3* KO compared to parental cells were identified using [9] and significantly differentially expressed genes (Padj < 0.05) are shown in red. The *Mbd1* gene is indicated by a dashed circle.

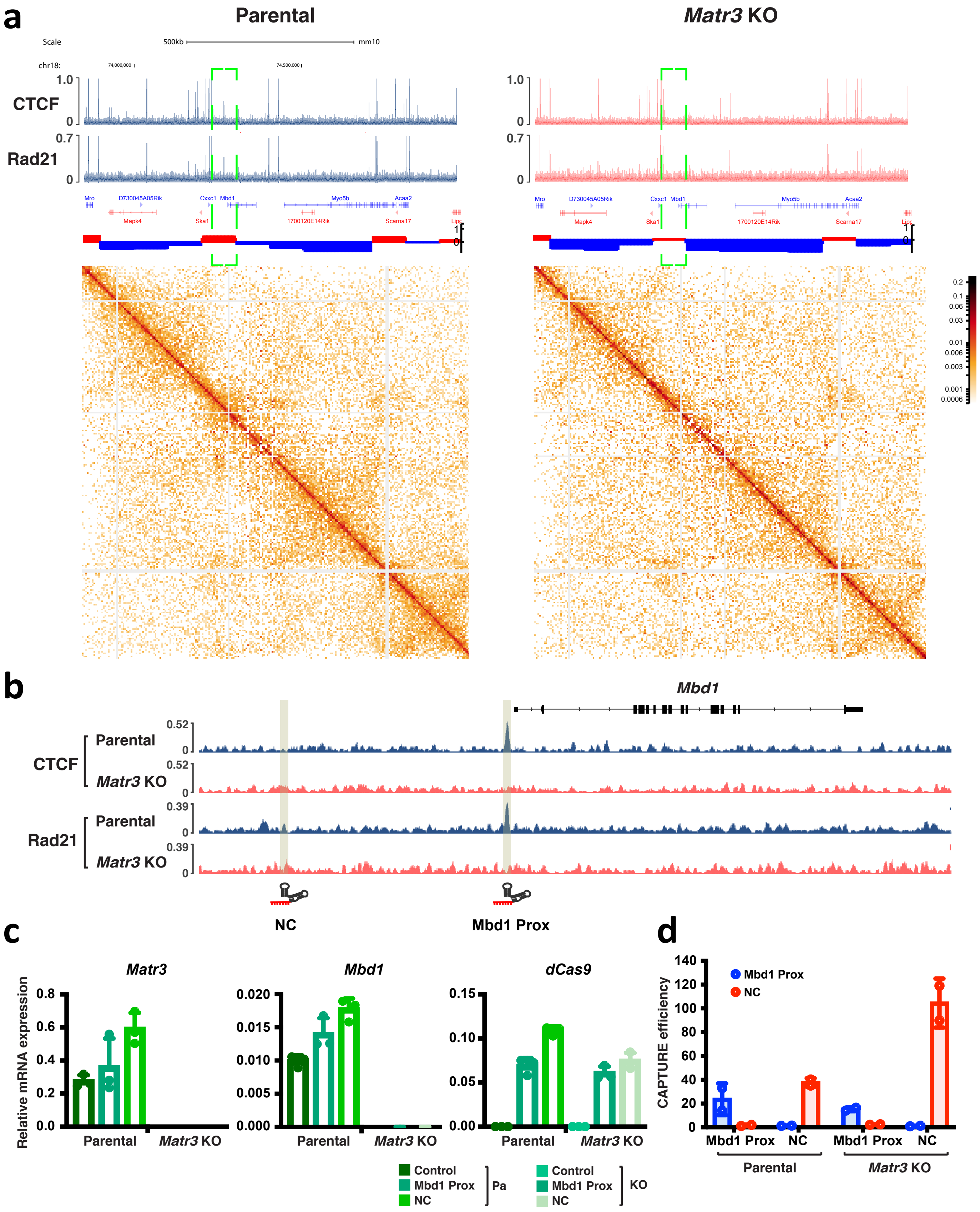
a
Parental
Matr3 KO
Scale
500kb
mm10
chr18:
CTCF
Rad21
b
Mbd1
CTCF
Parental
Matr3 KO
Rad21
Parental
Matr3 KO
NC
Mbd1 Prox
c
Matr3
Mbd1
dCas9
Relative mRNA expression
Parental
Matr3 KO
Control
Mbd1 Prox
NC
Pa
Control
Mbd1 Prox
NC
KO
d
CAPTURE efficiency
Mbd1 Prox
NC
Mbd1 Prox
NC
Mbd1 Prox
NC
Parental
Matr3 KO

Figure S4

Figure S4. Matr3 is directly involved in the regulation of gene expression at the Mbd1 locus. (a) Hi-C contact matrices at 5 kb resolution and compartments near the *Mbd1* gene. Dashed box indicates the CTCF/Rad21-occupied region upstream of *Mbd1*. (b) For the CAPTURE assay (Figure 5c), sgRNAs were designed to recruit biotinylated dCas9 to the putative regulatory element near *Mbd1* (Mbd1 Prox) occupied by CTCF and cohesin, and the adjacent upstream as a negative control (NC). (c) Quality control of gene expression after cell line derivation. Expression of Mbd1 or Matr3 was unaffected by dCas9/sgRNA targeting the respective cis regions (n = 3). (d) Comparable enrichment of targeted genomic regions was observed for both sgRNAs in parental and *Matr3* KO cells. Fold enrichment was calculated using ddCt method against a negative control ChIP primer (n = 2). Error bars represent mean ± 1 s.d. across 2–3 independent experiments. Source data are provided as a Source Data file.

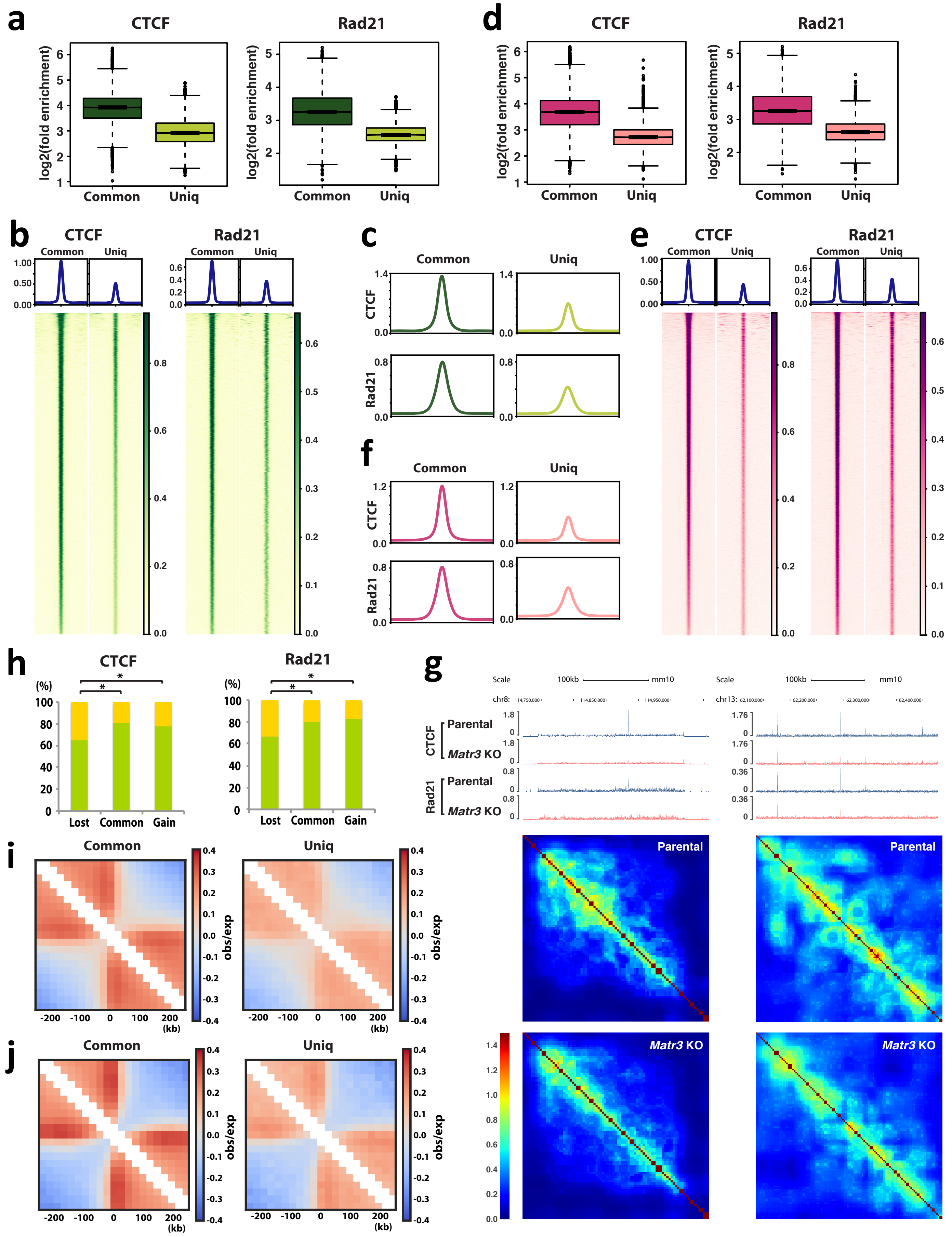
a
CTCF
Rad21
log2(fold enrichment)
Common
Uniq
d
b
c
e
f
Rad21
h
(%)
Lost
Common
Gain
g
Scale
100kb
mm10
chr8:
chr13:
Parental
Matr3 KO
i
j
obs/exp
(kb)

Figure S5

Figure S5. Low occupancy sites of CTCF and cohesin appear susceptible to developmental regulation and Matr3 loss. (a) Peak enrichment at altered sites (uniq) compared to maintained sites (common) as described in Figure 6d ($p < 2.2e-16$, $p < 2.2e-16$, respectively, by two-sided t test; Cohen's d = 1.42, Cohen's d = 1.15, respectively; n = 17437, n= 13381, n = 9073, n = 3131, respectively). (b-c) After classification of regions during differentiation (Figure 6c), enrichment scores per genomic region were calculated by [10] in each experiment and the results were confirmed in 2-3 independent experiments. (d) Peak enrichment at altered sites (uniq) compared to maintained sites (common) as described in Figure 6f ($p < 2.2e-16$, $p < 2.2e-16$, respectively, by two-sided t test; Cohen's d = 1.17, Cohen's d = 1.06, respectively; n = 25310, n = 5529, n = 8803, n = 3425, respectively). (e-f) After classification of regions in *Matr3* KO compared to parental cells (Figure 4d), enrichment scores per genomic region were calculated by [10] in each experiment and the results were confirmed in 2-3 independent experiments. (g) Examples of regions showing altered chromatin interaction loops with reduced CTCF and Rad21 binding in *Matr3* KO compared to control. (h) Percentage of classified ChIP-seq peaks (Figure 4d) in compartments A (green) and B (yellow). Higher fraction of sites with reduced occupancy of CTCF and Rad21 was in compartment B compared to the common or gained regions. Ratios of A/B are 1.86, 4.34, and 3.54 for CTCF, respectively, and 1.99, 4.03, and 4.77 for Rad21, respectively ($p < 1e-5$, $p < 1e-5$, $p < 1e-5$, $p < 1e-5$, by chi-square test, respectively). Interaction pile-up maps of Hi-C data at the boundaries defined by each set of CTCF (i) and Rad21 (j) ChIP-seq peaks indicate stronger insulation at regions maintained (common) than at altered sites (uniq) in *Matr3* KO cells. The results were

confirmed in 2-3 independent experiments. In box plots, the centre line represents the median, box limits show upper and lower quartiles, and whiskers extend to 1.5 × interquartile range.

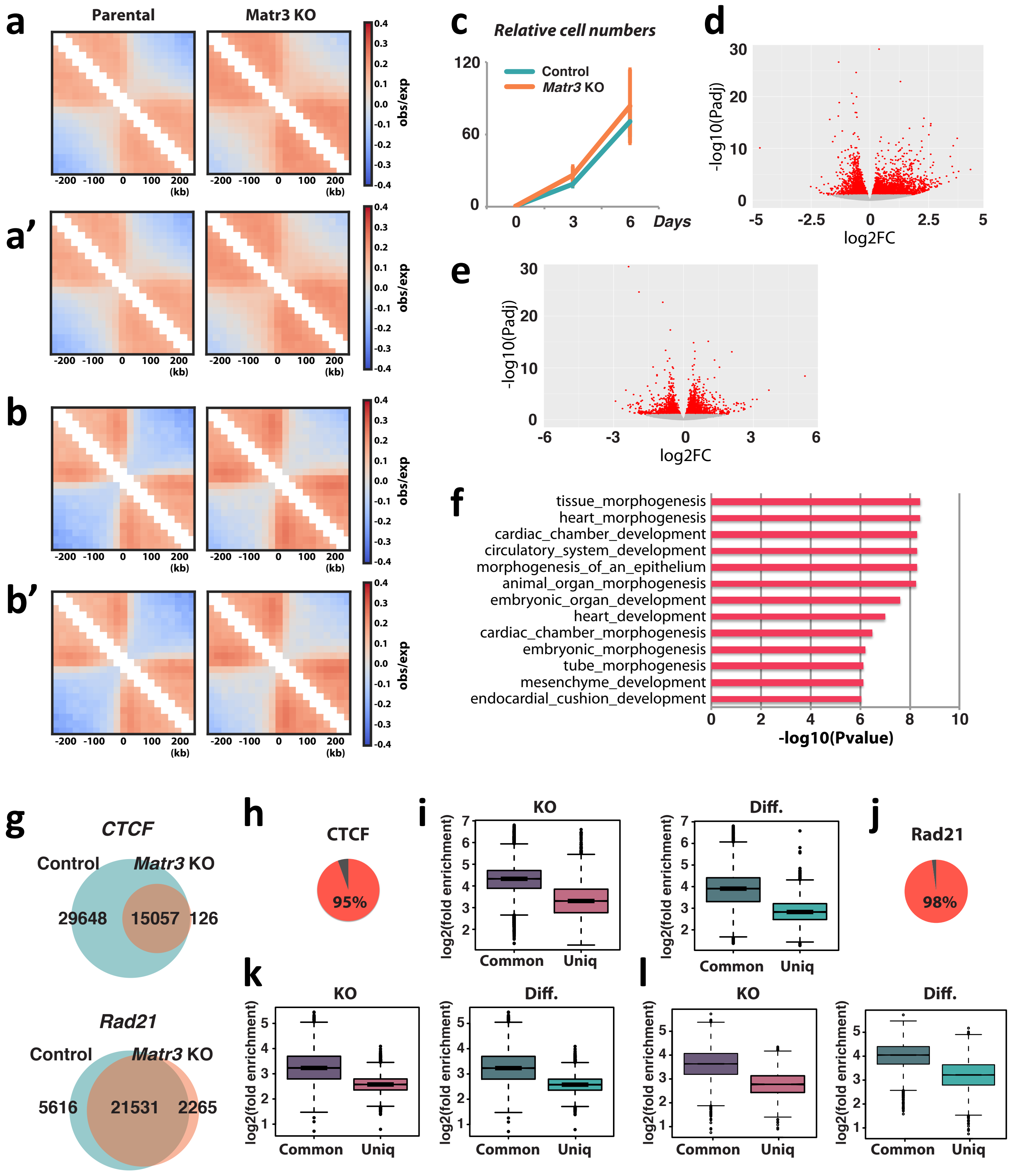
a
Parental
Matr3 KO
obs/exp
a'
b
b'
-200 -100 0 100 200
(kb)
c
Relative cell numbers
Control
Matr3 KO
Days
d
-log10(Padj)
log2FC
e
f
tissue_morphogenesis
heart_morphogenesis
cardiac_chamber_development
circulatory_system_development
morphogenesis_of_an_epithelium
animal_organ_morphogenesis
embryonic_organ_development
heart_development
cardiac_chamber_morphogenesis
embryonic_morphogenesis
tube_morphogenesis
mesenchyme_development
endocardial_cushion_development
-log10(Pvalue)
g
CTCF
Control
Matr3 KO
29648
15057
126
Rad21
5616
21531
2265
h
95%
i
KO
Diff.
log2(fold enrichment)
Common
Uniq
j
98%
k
l

Figure S6

Figure S6. *Matr3* KO affects ES cell differentiation and the low occupied sites of CTCF and cohesin appear susceptible to developmental regulation and loss of Matr3 in ES cells. (a-b') Interaction pile-up maps of Hi-C data at boundaries defined by altered CTCF (a-a') and Rad21 (b-b') ChIP-seq peaks in *Matr3* KO. (c) Cell proliferation was examined in control (n = 3) and four independent *Matr3* KO (n = 4) ES cell clones. Error bar, SEM. Differentially expressed genes in *Matr3* KO compared to control cells under uninduced (d) and differentiated (e) conditions. Significantly differentially expressed genes (Padj < 0.05) are shown in red. (f) Gene Ontology annotations for genes upregulated in *Matr3* KO ES cells during differentiation. (g) ChIP-seq datasets of CTCF and Rad21 were quantitatively compared[11]. The results of at least two independent experiments were combined to generate a more stringent peak list. (h) Percentage of the binding regions lost in *Matr3* KO compared to control ES cells in sites with reduced CTCF binding during differentiation. (i) Peak enrichment at altered CTCF sites (uniq) compared to maintained sites (common) in *Matr3* KO cells and during the differentiation (diff) process ($p < 2.2e-16$, $p < 2.2e-16$, respectively, by two-sided t test; Cohen's $d = 1.31$, Cohen's $d = 0.79$, respectively; $n = 14940$, $n= 25254$, $n = 33077$, $n = 7164$, respectively). (j) Percentage of regions with reduced Rad21 binding during differentiation in the binding sites lost in *Matr3* KO compared to control ES cells. (k-l) Peak enrichment at altered Rad21 sites (uniq) compared to maintained sites (common) in *Matr3* KO ES cells and during the differentiation (diff) process ($p < 2.2e-16$, $p < 2.2e-16$, $p < 2.2e-16$, $p < 2.2e-16$, respectively, by two-sided t test; Cohen's $d = 1.15$, Cohen's $d = 1.47$, Cohen's $d = 1.00$, Cohen's $d = 1.00$, respectively; $n = 21754$, $n= 5622$, $n = 8271$, $n = 19117$, $n = 21543$, $n= 5617$, $n = 8132$, $n = 19021$,

respectively). In box plots, the centre line represents the median, box limits show upper and lower quartiles, and whiskers extend to 1.5 × interquartile range.

TABLES

Table S1. GATA motifs are significantly enriched in newly opened regions of *Matr3* KO compared to parental cells. ATAC-seq peaks unique to *Matr3* KO compared to parental cells were used for motif analysis. The raw number and percentage of sequences used in the analysis and the natural logarithm of p-values calculated using the binomial distribution are shown.

Name	log p-value	# Target Sequences with Motif	% of Targets Sequences with Motif	# Background Sequences with Motif	% of Background Sequences with Motif
Gata4 (Zf)	-5.46E+02	1034	27.54%	4115.3	8.94%
Gata6 (Zf)	-5.33E+02	967	25.76%	3701.1	8.04%
Gata3 (Zf)	-5.29E+02	1276	33.99%	6097.6	13.25%
Gata2 (Zf)	-5.21E+02	823	21.92%	2786.2	6.05%
Gata1 (Zf)	-5.09E+02	773	20.59%	2518.2	5.47%
PU.1 (ETS)	-1.94E+02	489	13.03%	2227.6	4.84%
ETS1 (ETS)	-1.75E+02	769	20.48%	4712.9	10.24%

REFERENCES

1. Bauer, D. E., Canver, M. C. & Orkin, S. H. Generation of genomic deletions in mammalian cell lines via CRISPR/Cas9. *J. Vis. Exp.* 1–10 (2015). doi:10.3791/52118
2. Li, L. Q. *et al.* Ldb1-nucleated transcription complexes function as primary mediators of global erythroid gene activation. *Blood* **121**, 4575–4585 (2013).
3. Falk, M. *et al.* Heterochromatin drives compartmentalization of inverted and conventional nuclei. *Nature* **570**, 395–399 (2019).
4. Calandrelli, R., Wu, Q., Guan, J. & Zhong, S. GITAR: An Open Source Tool for Analysis and Visualization of Hi-C Data. *Genomics, Proteomics Bioinforma.* **16**, 365–372 (2018).
5. Serra, F. *et al.* Automatic analysis and 3D-modelling of Hi-C data using TADbit reveals structural features of the fly chromatin colors. *PLoS Comput. Biol.* **13**, 1–17 (2017).
6. Katz, Y., Wang, E. T., Airoldi, E. M. & Burge, C. B. Analysis and design of RNA sequencing experiments for identifying isoform regulation. *Nat. Methods* **7**, 1009–1015 (2010).
7. Liu, Z. *et al.* Single-cell transcriptomics reconstructs fate conversion from fibroblast to cardiomyocyte. *Nature* **551**, 100–104 (2017).
8. Robinson, M. D., McCarthy, D. J. & Smyth, G. K. edgeR: A Bioconductor package for differential expression analysis of digital gene expression data. *Bioinformatics* **26**, 139–140 (2009).
9. Love, M. I., Huber, W. & Anders, S. Moderated estimation of fold change and dispersion for RNA-seq data with DESeq2. *Genome Biol.* **15**, 1–21 (2014).
10. Ramírez, F. *et al.* deepTools2: a next generation web server for deep-sequencing data analysis. *Nucleic Acids Res.* **44**, W160–W165 (2016).
11. Shao, Z., Zhang, Y., Yuan, G. C., Orkin, S. H. & Waxman, D. J. MAnorm: A robust model for quantitative comparison of ChIP-Seq data sets. *Genome Biol.* **13**, (2012).
