# Supplementary material for: Inner nuclear protein Matrin-3 coordinates cell differentiation by stabilizing chromatin architecture
Source: Nat Commun. 2021 Oct 29;12:6241. doi: 10.1038/s41467-021-26574-4 (PMC8556400; doi:10.1038/s41467-021-26574-4)
Supplement: Supplementary file 3 — Reporting Summary [file 41467_2021_26574_MOESM3_ESM.pdf]

nature portfolio

Corresponding author(s): Stuart H Orkin

Last updated by author(s): Sep 19, 2021

Reporting Summary

Nature Portfolio wishes to improve the reproducibility of the work that we publish. This form provides structure for consistency and transparency in reporting. For further information on Nature Portfolio policies, see our Editorial Policies and the Editorial Policy Checklist.

Statistics

For all statistical analyses, confirm that the following items are present in the figure legend, table legend, main text, or Methods section.

n/a	Confirmed	
☐	☒	The exact sample size (*n*) for each experimental group/condition, given as a discrete number and unit of measurement
☐	☒	A statement on whether measurements were taken from distinct samples or whether the same sample was measured repeatedly
☐	☒	The statistical test(s) used AND whether they are one- or two-sided<br>*Only common tests should be described solely by name; describe more complex techniques in the Methods section.*
☒	☐	A description of all covariates tested
☐	☒	A description of any assumptions or corrections, such as tests of normality and adjustment for multiple comparisons
☐	☒	A full description of the statistical parameters including central tendency (e.g. means) or other basic estimates (e.g. regression coefficient) AND variation (e.g. standard deviation) or associated estimates of uncertainty (e.g. confidence intervals)
☐	☒	For null hypothesis testing, the test statistic (e.g. *F*, *t*, *r*) with confidence intervals, effect sizes, degrees of freedom and *P* value noted<br>*Give P values as exact values whenever suitable.*
☒	☐	For Bayesian analysis, information on the choice of priors and Markov chain Monte Carlo settings
☒	☐	For hierarchical and complex designs, identification of the appropriate level for tests and full reporting of outcomes
☐	☒	Estimates of effect sizes (e.g. Cohen's *d*, Pearson's *r*), indicating how they were calculated

Our web collection on statistics for biologists contains articles on many of the points above.

Software and code

Policy information about availability of computer code

Data collection	Data collection No software was used.
Data analysis	pairtools (https://github.com/mirnylab/pairtools; v0.2.2), cooltools (https://github.com/mirnylab/cooltools; v0.2.0), HiC-Pro (v2.11.1), cooler package (v0.8.6), juicer tools (v1.7.5), HOMER (v4.9), HiCtool package (https://github.com/Zhong-Lab-UCSD/HiCtool; v2.1), HIFI package (https://github.com/BlanchetteLab/HIFI; v1.0.0), SEQUEST (v28 rev 13), Spectrum Mill software package v6.0 prerelease, Cutadapt (v1.14), Bowtie2 (v2.2.9), MACS2 (v2.1.1.20160309), MAnorm (v1.1.4), STAR (v2.5.4a), HTseq (v0.9.1), edgeR (v3.28.1), DESeq2 (v1.26.0), MISO (v0.5.4), HISAT2 (v2.1.0), https://ccb.jhu.edu/software/stringtie/dl/prepDE.py, SEEK (http://seek.princeton.edu/; v1), Picard Tools (http://broadinstitute.github.io/picard/; v2.8.0), Trimmomatic (v0.36), samtools (v1.3.1), HiCCUPS (v1.6)

For manuscripts utilizing custom algorithms or software that are central to the research but not yet described in published literature, software must be made available to editors and reviewers. We strongly encourage code deposition in a community repository (e.g. GitHub). See the Nature Portfolio guidelines for submitting code & software for further information.

Data

Policy information about availability of data

All manuscripts must include a data availability statement. This statement should provide the following information, where applicable:

- Accession codes, unique identifiers, or web links for publicly available datasets
- A description of any restrictions on data availability
- For clinical datasets or third party data, please ensure that the statement adheres to our policy

Hi-C, ChIP-seq, ATAC-seq, and RNA-seq data sets used this study are available in the GEO database, under accession number GSE181234 [https://www.ncbi.nlm.nih.gov/geo/query/acc.cgi?acc=GSE181234].

Field-specific reporting

Please select the one below that is the best fit for your research. If you are not sure, read the appropriate sections before making your selection.

[x] Life sciences [ ] Behavioural & social sciences [ ] Ecological, evolutionary & environmental sciences

For a reference copy of the document with all sections, see nature.com/documents/nr-reporting-summary-flat.pdf

Life sciences study design

All studies must disclose on these points even when the disclosure is negative.

Sample size	Sample sizes was chosen based on published data using similar approaches. https://pubmed.ncbi.nlm.nih.gov/32817427/; https://pubmed.ncbi.nlm.nih.gov/28841410/
Data exclusions	No data were excluded.
Replication	All experiments were reliably repeated at least two times.
Randomization	Experimental groups of cells were assigned according to their genotype.
Blinding	Imaging data collection was made blinded to group allocation. Blinding was not relevant to the genomic data because investigator's bias would not affect the data collection. Data analysis was not blinded because it is strictly quantitative.

Reporting for specific materials, systems and methods

We require information from authors about some types of materials, experimental systems and methods used in many studies. Here, indicate whether each material, system or method listed is relevant to your study. If you are not sure if a list item applies to your research, read the appropriate section before selecting a response.

Materials & experimental systems

n/a	Involved in the study	
[ ]	[x]	Antibodies
[ ]	[x]	Eukaryotic cell lines
[x]	[ ]	Palaeontology and archaeology
[x]	[ ]	Animals and other organisms
[x]	[ ]	Human research participants
[x]	[ ]	Clinical data
[x]	[ ]	Dual use research of concern

Methods

n/a	Involved in the study	
[ ]	[x]	ChIP-seq
[x]	[ ]	Flow cytometry
[x]	[ ]	MRI-based neuroimaging

Antibodies

Antibodies used	Esco2 (bethyl, A301-689A, immunoblot, 1:1000), Matr3 (Abcam, ab84422, immunoblot, 1:1000; immunofluorescence, 1:100), CTCF (Abcam, ab70303, immunoblot, 1:1000), Rad21 (Abcam, ab992, immunoblot, 1:1000), Smc3 (Abcam, ab9263, immunoblot, 1:1000), Mbd1 (Abcam, ab187734, immunoblot, 1:1000), H3K4me1 (Abcam, ab8895), H3K27ac (Abcam, ab4729), H3K36me3 (Abcam, ab9050), Matr3 (Santa CruzBiotechnology, sc-81318, immunoblot, 1:200), HP1α (Abcam, ab203432, immunofluorescence, 1:100), Histone H3 (Abcam, ab1791), Smc1a (Abcam, ab140493).
Validation	anti-ESCO2 antibody has been used for immunoblotting of mouse oocytes (PMID: 32714760).<br>Matr3 antibody (ab84422) recognizes mouse and human MATR3 proteins; Application: immunoblotting, immunofluorescence, immunoprecipitation (https://www.abcam.com/matrin-3-antibody-ab84422.html; PMID: 27733621; PMID: 25119036).<br>CTCF antibody recognizes mouse and human CTCF proteins; Application: immunoblotting, immunoprecipitation (https://www.abcam.com/ctcf-antibody-ab70303.html).<br>Rad21 antibody recognizes mouse and human RAD21 proteins; Application: immunoblotting, immunoprecipitation (https://www.abcam.com/rad21-antibody-ab992.html).<br>Smc3 antibody recognizes mouse and human SMC3 proteins; Application: immunoblotting (https://www.abcam.com/smc3-antibody-ab9263.html).<br>Mbd1 antibody recognizes mouse MBD1 protein; Application: immunoblotting (http://www.abcam.com/MBD1-antibody-7A3C12A9-ab187734.html).<br>H3K4me1 antibody recognizes mouse mono-methylated Lysine 4 of histone H3; Application: ChIP (https://www.abcam.com/histone-h3-mono-methyl-k4-antibody-chip-grade-ab8895.html).<br>H3K27ac antibody recognizes mouse histone H3 (acetyl K27); Application: ChIP (https://www.abcam.com/histone-h3-acetyl-k27-antibody-chip-grade-ab4729.html).<br>H3K36me3 antibody recognizes mouse Histone H3 (tri methyl K36); Application: ChIP (PMID: 23160351).

Matr3 antibody (sc-81318) recognizes mouse MATR3 proteins; Application: immunoblotting (https://www.scbt.com/p/matrin-3-antibody-2539c3a).
HP1α antibody recognizes mouse HP1α proteins; Application: immunofluorescence (PMID: 31391587).
Histone H3 antibody recognizes mouse Histone H3; Application: immunoblotting (https://www.abcam.com/histone-h3-antibody-nuclear-marker-and-chip-grade-ab1791.html).
Smc1a antibody recognizes human and mouse SMC1A proteins; Application: immunoblotting, immunoprecipitation (https://www.abcam.com/smc1a-antibody-ab140493.html).

Eukaryotic cell lines

Policy information about cell lines

Cell line source(s)	MEL cells, mouse embryonic stem cells, and G1ER were derived in house. 293T cells were purchased from ATCC.
Authentication	Cell lines were not authenticated.
Mycoplasma contamination	All cell lines were tested negative for mycoplasma contamination by PCR.
Commonly misidentified lines (See ICLAC register)	None

ChIP-seq

Data deposition

- [x] Confirm that both raw and final processed data have been deposited in a public database such as GEO.
- [x] Confirm that you have deposited or provided access to graph files (e.g. BED files) for the called peaks.

Data access links
May remain private before publication.

ChIP-seq data sets used this study are available in the GEO database, under accession number GSE181231 (token: clavgswurpgfbup).

Files in database submission

MEL_parental_H3K27Ac_rep1.peaks MEL_parental_H3K27Ac_rep1.fastq.gz
MEL_Matr3KO_H3K27Ac_rep1.peaks MEL_Matr3KO_H3K27Ac_rep1.fastq.gz
MEL_parental_H3K27Ac_rep2.peaks MEL_parental_H3K27Ac_rep2.fastq.gz
MEL_Matr3KO_H3K27Ac_rep2.peaks MEL_Matr3KO_H3K27Ac_rep2.fastq.gz
MEL_parental_H3K4me1.peaks MEL_parental_H3K4me1.fastq.gz
MEL_Matr3KO_H3K4me1.peaks MEL_Matr3KO_H3K4me1.fastq.gz
MEL_parental_H3K36me3.peaks MEL_parental_H3K36me3.fastq.gz
MEL_Matr3KO_H3K36me3.peaks MEL_Matr3KO_H3K36me3.fastq.gz
MEL_parental_CTCF_rep1.peaks MEL_parental_CTCF_rep1.fastq.gz
MEL_Matr3KO_CTCF_rep1.peaks MEL_Matr3KO_CTCF_rep1.fastq.gz
MEL_diff_CTCF_rep1.peaks MEL_diff_CTCF_rep1.fastq.gz
MEL_parental_CTCF_rep2.peaks MEL_parental_CTCF_rep2.fastq.gz
MEL_Matr3KO_CTCF_rep2.peaks MEL_Matr3KO_CTCF_rep2.fastq.gz
MEL_diff_CTCF_rep2.peaks MEL_diff_CTCF_rep2.fastq.gz
MEL_parental_Rad21_rep1.peaks MEL_parental_Rad21_rep1.fastq.gz
MEL_Matr3KO_Rad21_rep1.peaks MEL_Matr3KO_Rad21_rep1.fastq.gz
MEL_diff_Rad21_rep1.peaks MEL_diff_Rad21_rep1.fastq.gz
MEL_parental_Rad21_rep2.peaks MEL_parental_Rad21_rep2.fastq.gz
MEL_Matr3KO_Rad21_rep2.peaks MEL_Matr3KO_Rad21_rep2.fastq.gz
MEL_diff_Rad21_rep2.peaks MEL_diff_Rad21_rep2.fastq.gz
ESC_WT_CTCF_rep1.peaks ESC_WT_CTCF_rep1.fastq.gz
ESC_Matr3KO_CTCF_rep1.peaks ESC_Matr3KO_CTCF_rep1.fastq.gz
ESC_diff_CTCF_rep1.peaks ESC_diff_CTCF_rep1.fastq.gz
ESC_WT_CTCF_rep2.peaks ESC_WT_CTCF_rep2.fastq.gz
ESC_Matr3KO_CTCF_rep2.peaks ESC_Matr3KO_CTCF_rep2.fastq.gz
ESC_WT_Rad21_rep1.peaks ESC_WT_Rad21_rep1.fastq.gz
ESC_Matr3KO_Rad21_rep1.peaks ESC_Matr3KO_Rad21_rep1.fastq.gz
ESC_diff_Rad21_rep1.peaks ESC_diff_Rad21_rep1.fastq.gz
ESC_WT_Rad21_rep2.peaks ESC_WT_Rad21_rep2.fastq.gz
ESC_Matr3KO_Rad21_rep2.peaks ESC_Matr3KO_Rad21_rep2.fastq.gz
ESC_diff_Rad21_rep2.peaks ESC_diff_Rad21_rep2.fastq.gz

Genome browser session
(e.g. UCSC)

No longer applicable.

Methodology

Replicates	In general, ChIP-seq experiments were performed in two replicates. The number of replicates is indicated in each file name.

Sequencing depth	MEL_parental_H3K27Ac_rep1.fastq.gz 49635339 39809720 50 SE<br>MEL_Matr3KO_H3K27Ac_rep1.fastq.gz 54293644 38154809 50 SE<br>MEL_parental_H3K27Ac_rep2.fastq.gz 39257977 34139901 75 SE<br>MEL_Matr3KO_H3K27Ac_rep2.fastq.gz 35693418 31067304 75 SE<br>MEL_parental_H3K4me1.fastq.gz 40119370 33182323 50 SE<br>MEL_Matr3KO_H3K4me1.fastq.gz 42154639 35970347 50 SE<br>MEL_parental_H3K36me3.fastq.gz 27880264 24751568 60 SE<br>MEL_Matr3KO_H3K36me3.fastq.gz 31773461 28018031 60 SE<br>MEL_parental_CTCF_rep1.fastq.gz 41919955 35336313 50 SE<br>MEL_Matr3KO_CTCF_rep1.fastq.gz 49842545 41806988 50 SE<br>MEL_diff_CTCF_rep1.fastq.gz 34233703 29034877 75 SE<br>MEL_parental_CTCF_rep2.fastq.gz 32618486 27440267 75 SE<br>MEL_Matr3KO_CTCF_rep2.fastq.gz 26010972 22532677 75 SE<br>MEL_diff_CTCF_rep2.fastq.gz 29328292 25034028 75 SE<br>MEL_parental_Rad21_rep1.fastq.gz 89550697 70017239 50 SE<br>MEL_Matr3KO_Rad21_rep1.fastq.gz 56843870 46416665 50 SE<br>MEL_diff_Rad21_rep1.fastq.gz 44579106 37322083 75 SE<br>MEL_parental_Rad21_rep2.fastq.gz 29857729 25336191 75 SE<br>MEL_Matr3KO_Rad21_rep2.fastq.gz 33065405 27776634 75 SE<br>MEL_diff_Rad21_rep2.fastq.gz 33959247 28385696 75 SE<br>ESC_WT_CTCF_rep1.fastq.gz 52962758 38253737 75 SE<br>ESC_Matr3KO_CTCF_rep1.fastq.gz 19796597 16726697 75 SE<br>ESC_diff_CTCF_rep1.fastq.gz 42928611 35369006 75 SE<br>ESC_WT_CTCF_rep2.fastq.gz 18469472 15551653 75 SE<br>ESC_Matr3KO_CTCF_rep2.fastq.gz 18995626 15714465 75 SE<br>ESC_WT_Rad21_rep1.fastq.gz 30639560 25830619 75 SE<br>ESC_Matr3KO_Rad21_rep1.fastq.gz 12270131 10554548 75 SE<br>ESC_diff_Rad21_rep1.fastq.gz 26915366 21502895 75 SE<br>ESC_WT_Rad21_rep2.fastq.gz 20820653 17866834 75 SE<br>ESC_Matr3KO_Rad21_rep2.fastq.gz 24718305 20588654 75 SE<br>ESC_diff_Rad21_rep2.fastq.gz 37093623 29764859 75 SE
Antibodies	CTCF (Abcam, ab70303), Rad21 (Abcam, ab992), H3K4me1 (Abcam, ab8895), H3K27ac (Abcam, ab4729), H3K36me3 (Abcam, ab9050)
Peak calling parameters	Bowtie2 was used to align sequencing reads with the default parameters. Peaks were called using MACS2 and significant enrichment regions were determined by -q 0.01.
Data quality	Data quality was verified with FastQC.
Software	FASTQC 0.11.3, Bowtie 2.2.9, Samtools 1.3.1, Picard 2.8.0, MACS 2.1.1,MAnorm, HISAT2, https://ccb.jhu.edu/software/stringtie/dl/prepDE.py, SEEK (http://seek.princeton.edu/)
