# Supplementary figures and images for: Inner nuclear protein Matrin-3 coordinates cell differentiation by stabilizing chromatin architecture
Source: Nat Commun. 2021 Oct 29;12:6241. doi: 10.1038/s41467-021-26574-4 (PMC8556400; doi:10.1038/s41467-021-26574-4)

Figure 1.

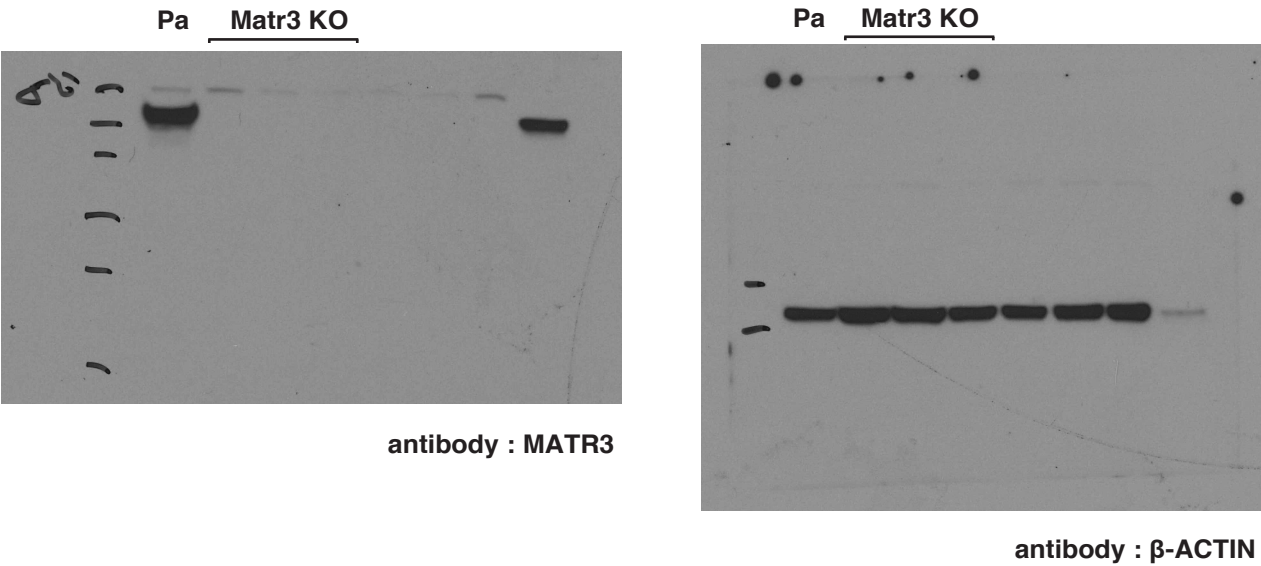

Figure 4A.

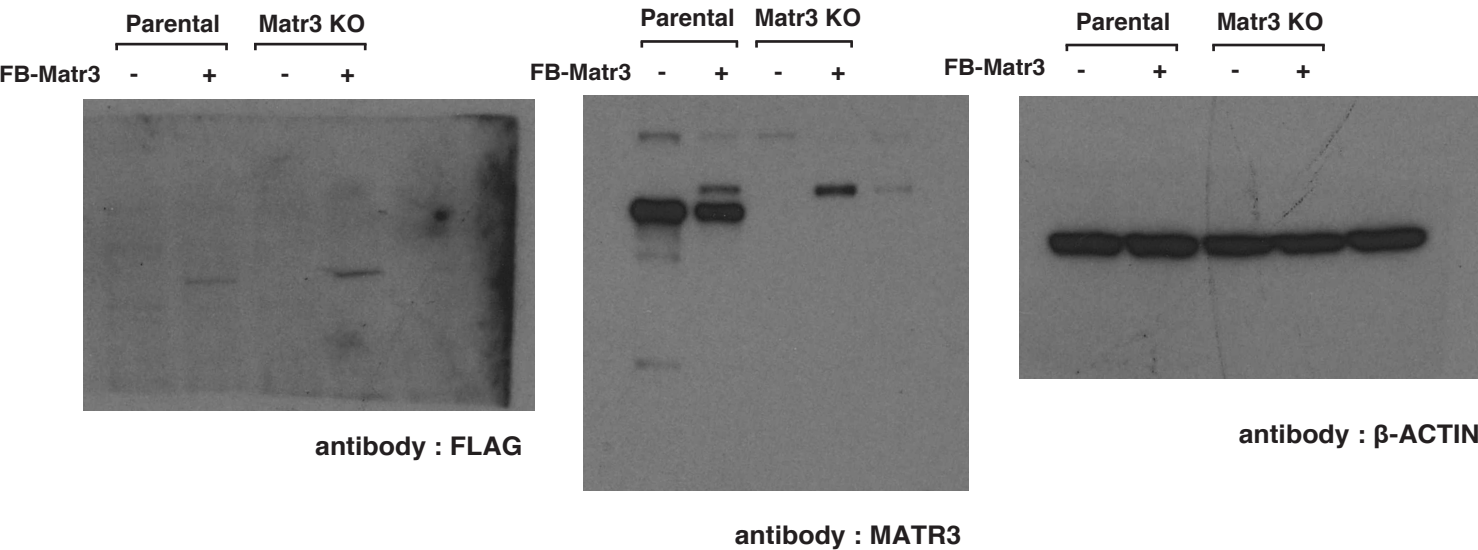

**Figure 4B.**

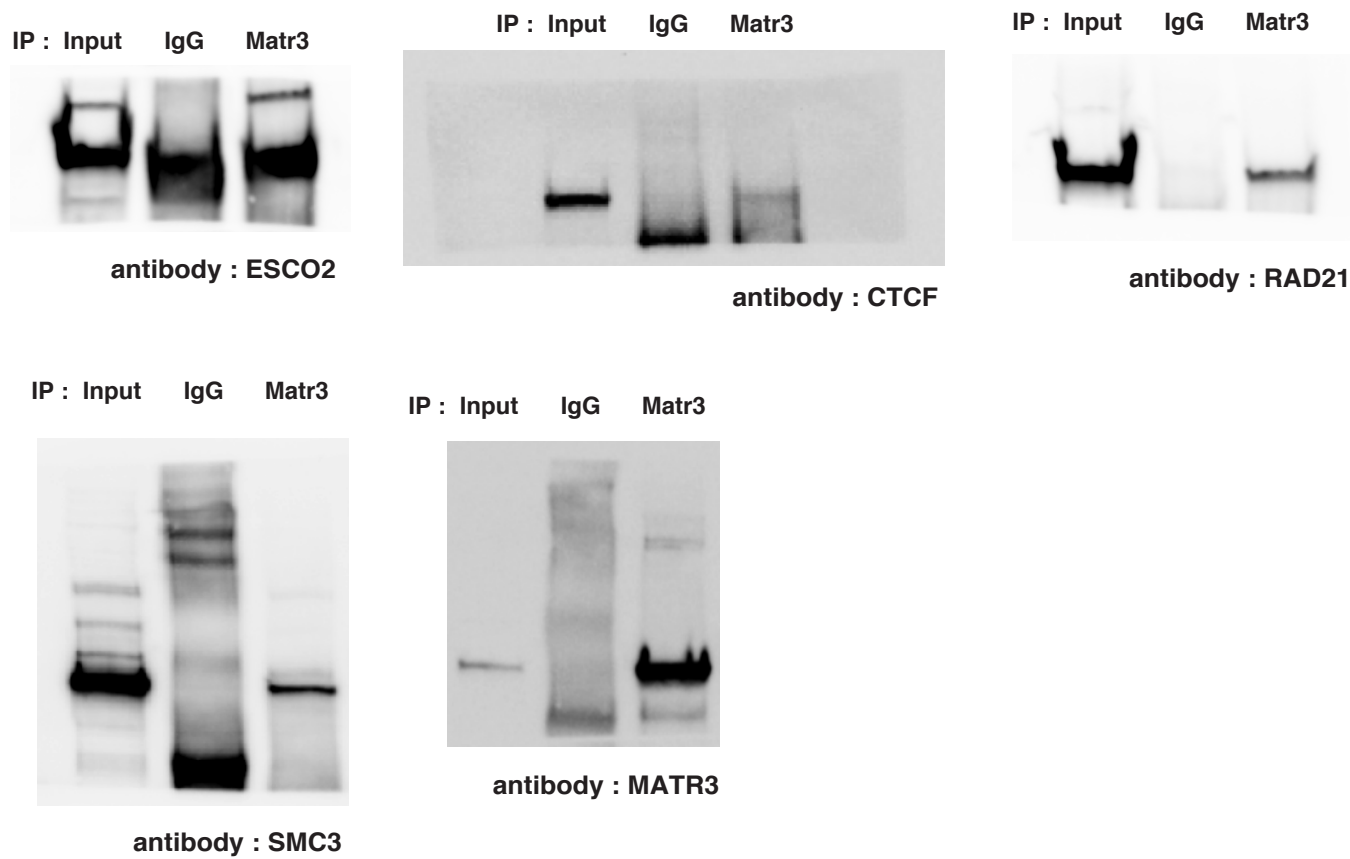

**Figure 5A.**

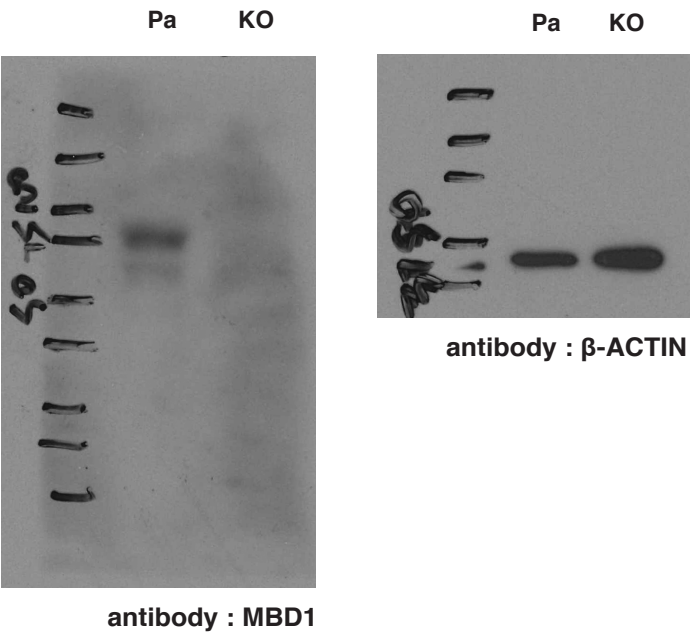

Figure 5D.

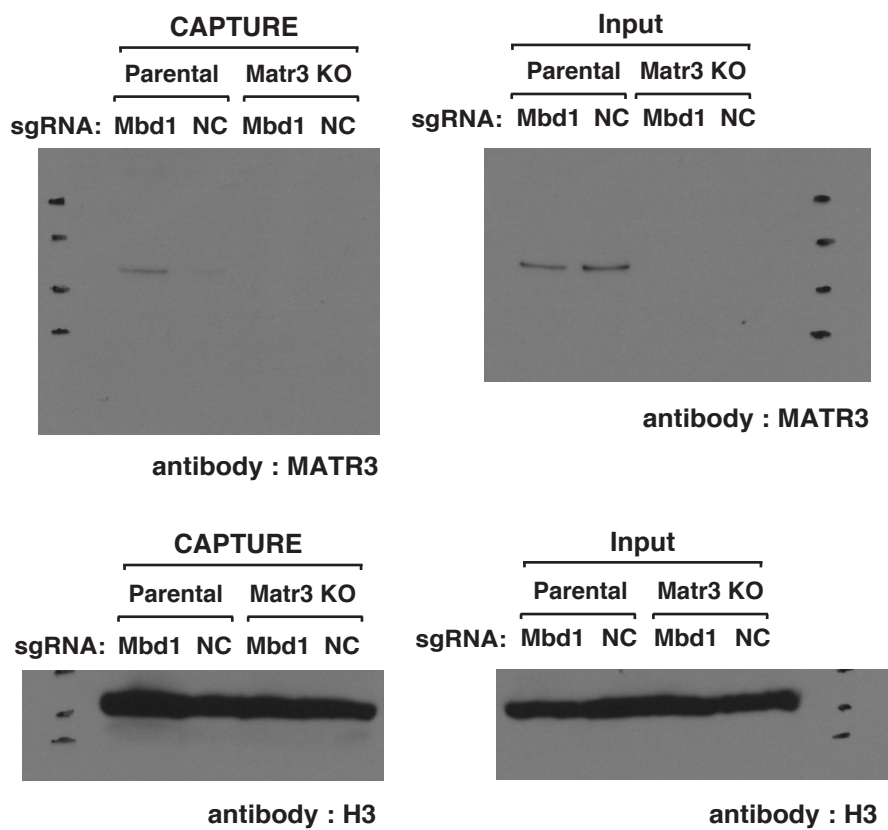

Figure 5H.

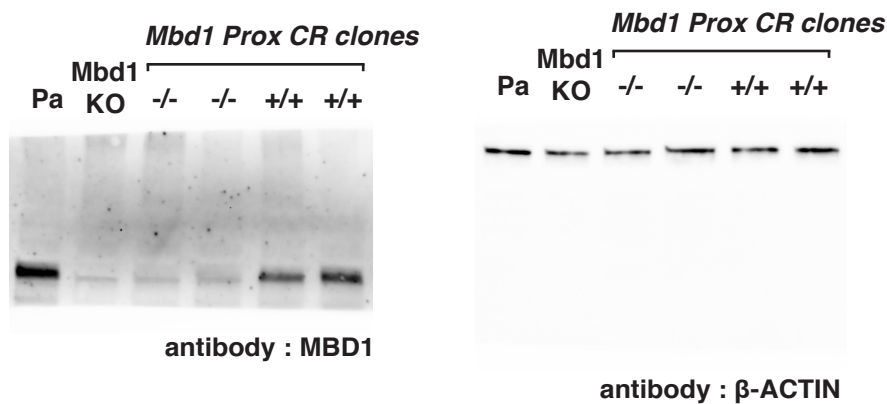

Figure S1C.

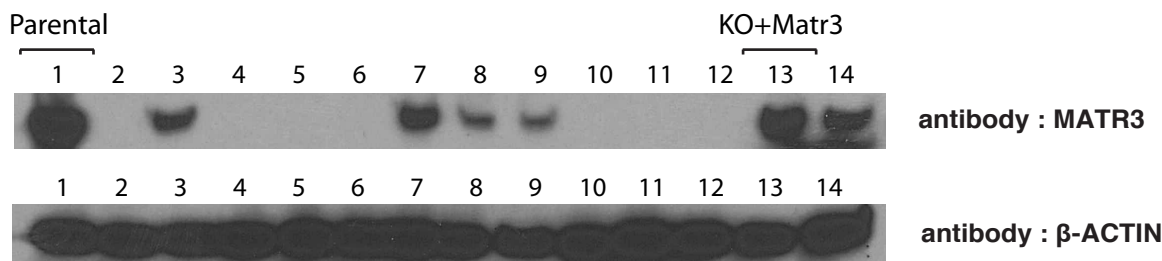

Supplement: Supplementary file 4 — Source data [file 41467_2021_26574_MOESM4_ESM.zip › NCOMMS-21-05375B/Source Data 2.pdf]
